# Foreword

**DOI:** 10.1186/1472-6963-15-S3-I1

**Published:** 2015-11-06

**Authors:** Fahdi Dkhimi, Werner Soors, Bart Criel

**Affiliations:** 1 Unit of Equity & Health, Department of Public Health, Institute of Tropical Medicine, Antwerp, Belgium

## 

Universal Health Coverage (UHC) is increasingly gaining centre stage in the post 2015 international development agenda [1]. After years of debate [2-4], a consensus has gradually emerged that UHC is "an affordable dream" [5] that can have a significant impact on people's individual and collective wellbeing. Comprehensive monitoring frameworks to measure progress towards UHC are being developed [6]. Eventually, UHC is seen as a vehicle to bring about health equity [7].

The concern for equity in health is of course all but new [8]. However, equity as a moral imperative has not always been a prime concern in health financing [9]. Back in the 1980s, efficiency was the dominant driver of health financing reforms under structural adjustment. Corrective measures such as fee exemptions for indigents followed, but rarely proved to be effective [10-12]. To this added a growing body of evidence on the negative impact of user fees on health equity [13-16]. It is thus no wonder that the UHC debate at the beginning of the 21^st ^century renewed and boosted interest in equity [17-20].

Designing and implementing equity-oriented strategies remains, however, a key challenge in low- and middle-income countries [21]. Policymakers and implementers increasingly demand practical insights and knowledge [22], on which the otherwise copious UHC literature is particularly silent. Jim Yong Kim, President of the World Bank, recently made a case for "science of delivery", i.e. a science of implementation and execution [23]. We are witnessing a shift from the 'what' and 'why' questions to the 'how' question: how to steer complex policy processes in a way that enhances equity in health [24]. Over recent years, many policymakers in sub-Saharan Africa have opted for exemption policies to lower financial barriers to health care. In West Africa, this has led to a range of user-fee exemptions for vulnerable populations (e.g. children under-5, pregnant women), life-saving interventions (e.g. C-sections) or a combination of both (e.g. malaria treatment for under-5s).

To date, research investigating fee exemption policies by and large remains confined to their (quantitative) impact on utilisation rates, the most frequently used proxy for access to health care [25]. This type of evidence is obviously relevant, but hardly provides insight in how and why exemption policies work (or not). Limited evidence on the mechanisms of success and failure shows mixed results [26-31], with two conclusions systematically surfacing: the impact of fee exemptions is highly context-dependent; its understanding requiring in-depth exploration of both intended and unintended effects.

This special issue on fee exemption policies in West Africa couldn't be more timely. In the papers presented in this issue, the authors present results from research conducted by a range of teams led by Valéry Ridde and Jean-Pierre Olivier de Sardan in Mali, Niger and Burkina Faso over the past 5 to 10 years. The question of implementation is central in all the contributions.

Exploring policy implementation requires a certain level of flexibility, adapting and combining research methods in a way that enables the capture of complex social processes. This is precisely what the contributors to this special issue did. They present interesting examples of research endeavours, conducted simultaneously or successively, borrowing from various disciplines: history, sociology, political science, anthropology, and of course public health. The added value of such a blended approach - allowing for in-depth analysis of social, political, economic and cultural dimensions of public policies - cannot be underestimated.

We are confident that this issue will feed into the current debates on how to develop health equity intervention research [32]. More specifically, it will help to improve evaluation methodologies that capture contextual and other critical influences to understand what works to make significant progress towards UHC and how and why it does. Ultimately, such comprehensive knowledge can pave the way for transformative policies [33,34], a necessary condition for health equity.

## Declarations

This article has been published as part of *BMC Health Services *Research Volume 15 Supplement 3, 2015: User Fee Exemption Policies. The full contents of the supplement are available online at http://www.biomedcentral.com/bmchealthservres/supplements/15/S3.
